# Chili-supplemented food decreases glutathione-*S*-transferase activity in *Drosophila melanogaster* females without a change in other parameters of antioxidant system

**DOI:** 10.1080/13510002.2022.2123884

**Published:** 2022-10-06

**Authors:** Uliana V. Semaniuk, Dmytro V. Gospodaryov, Olha M. Strilbytska, Alicja Z. Kucharska, Anna Sokół-Łętowska, Nadia I. Burdyliuk, Kenneth B. Storey, Maria M. Bayliak, Oleh Lushchak

**Affiliations:** a Department of Biochemistry and Biotechnology, Vasyl Stefanyk Precarpathian National University, Ivano-Frankivsk, Ukraine; b Department of Fruit, Vegetable and Plant Nutraceutical Technology, Wrocław University of Environmental and Life Sciences, Wrocław, Poland; c Institute of Biochemistry, Carleton University, Ottawa, Canada; d Research and Development University, Ivano-Frankivsk, Ukraine

**Keywords:** Chili powder, antioxidant enzymes, sex dependence, oxidative stress indices, thiol-containing compounds, fruit fly

## Abstract

**Objectives:**

Many plant-derived anti-aging preparations influence antioxidant defense system. Consumption of food supplemented with chili pepper powder was found to extend lifespan in the fruit fly, *Drosophila melanogaster*. The present study aimed to test a connection between life-extending effect of chili powder and antioxidant defense system of *D. melanogaster*.

**Methods:**

Flies were reared for 15 days in the mortality cages on food with 0% (control), 0.04%, 0.12%, 0.4%, or 3% chili powder. Antioxidant and related enzymes, as well as oxidative stress indices were measured.

**Results:**

Female flies that consumed chili-supplemented food had a 40–60% lower glutathione-*S*-transferase (GST) activity as compared with the control cohort. Activity of superoxide dismutase (SOD) was about 37% higher in males that consumed food with 3% chili powder in comparison with the control cohort. Many of the parameters studied were sex-dependent.

**Conclusions:**

Consumption of chili-supplemented food extends lifespan in fruit fly cohorts in a concentration- and gender-dependent manner. However, this extension is not mediated by a strengthening of antioxidant defenses. Consumption of chili-supplemented food does not change the specific relationship between antioxidant and related enzymes in *D. melanogaster*, and does not change the linkage of the activities of these enzymes to fly gender.

## 1. Introduction

Beneficial effects of medicinal plant preparations on animal health are often associated with their secondary metabolites, that are mostly phenol-containing compounds. Special attention is paid to the antioxidant properties of plant phenols as well as to their ability to directly or indirectly activate cellular antioxidant systems [1]. The activation occurs predominantly by influencing specific transcription factors via direct interaction with them or via affecting their posttranscriptional modifications, crucial for activation or inhibition [1].

It was recently shown that capsaicin, a derivative of the phenol-containing compound, vanillylamine and a pungent principle component of chili pepper, prolongs lifespan in the fruit fly, *Drosophila melanogaster*, model [2]. We have conducted a complex study [3] to find whether the same capability to extend lifespan is attributable to chili powder, and not only to pure capsaicin. As in the above study of capsaicin effects [2], we used *D. melanogaster* as a tractable model, very suitable for a quick and inexpensive study.

Many plant-derived life-prolonging preparations influence antioxidant defense systems [4]. On one hand, this could be a side effect, that is not directly connected with a mechanism of the lifespan extension. Indeed, many signaling pathways that control cell senescence also have parallel effects on the activity of the antioxidant defense system. On the other hand, lifespan extension due to activation of antioxidant defenses is in good agreement with the free radical theory of aging, which is, despite a number of controversies, still accepted by many researchers [5–8].

**Figure 1.**
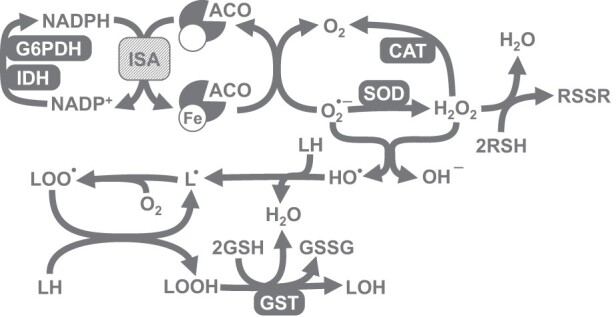
Explanation of the set of parameters measured in the study. Different types of reactive oxygen species (ROS), namely superoxide anion-radical, hydrogen peroxide, and hydroxyl radical, may hit various targets. Iron-sulfur (Fe-S) clusters present in a number of enzymes, including cytosolic and mitochondrial aconitase (ACO), are a well-established target of superoxide. Enzymes that reduce NADP (e.g. G6PDH and IDH) may provide NADPH for thioredoxin reductases or for the synthesis of Fe-S clusters by Fe-S cluster assembly (ISA) machinery. All types of ROS are able to oxidize thiol-containing compounds (RSH) and lipids, yielding disulfides (RSSR) and lipid peroxides (LOOH), respectively. SOD and catalase (CAT) convert ROS into less toxic species, whereas glutathione-*S*-transferase (GST) detoxifies lipid peroxides, preventing peroxidation chain reaction.

The present study is focused on the influence of chili-supplemented food on the activity of the antioxidant defense system of *D. melanogaster*. We have chosen a number of markers that would allow us to comprehensively evaluate the operation of antioxidant defenses. In particular, we measured the activities of first-line antioxidant enzymes, catalase and superoxide dismutase (SOD). Other important enzymes, that support the operation of multiple cellular peroxidases, are glucose 6-phosphate dehydrogenase (G6PDH) and isocitrate dehydrogenase (IDH). These enzymes reduce nicotinamide adenine dinucleotide phosphate (NADP^+^), yielding its protonated form, NADPH (Figure 1). Furthermore, NADPH is used for the reduction of thiol-containing antioxidants (e.g. glutathione, thioredoxin, and glutaredoxin) that in turn are oxidized by peroxidases. The expression of both NADPH-reducing enzymes was shown to be regulated by the transcription factors responsible for antioxidant defense [9]. We also measured glutathione-*S*-transferase (GST) activity. In most cases, GST allows conjugation of oxidized molecules, such as lipids, with glutathione, and their further excretion from the organism. Among oxidative stress indices, we chose low- and high-molecular mass thiol-containing compounds, lipid peroxides, carbonylated proteins, and the activity of aconitase, which contains superoxide-sensitive iron-sulfur clusters [10].

## 2. Materials and methods

### 2.1. Fly husbandry

Fruit flies of *Canton S* line were cultured in standard medium containing 5% sucrose, 5% yeast, 6% cornmeal, 1% agar, 0.18% methyl 4-hydroxyparabenzoic acid (methylparaben), and 0.6% propionic acid, 25°C, 60% relative humidity and 12h:12 h light/dark cycle. Newly eclosed flies were transferred to fresh medium, where they were kept during four days until the beginning of the experiment. Right before the beginning of the experiment flies were separated by sex under short (approx. 10 min) carbon dioxide anaesthesia and put into mortality cages at a density of approx. 150 individuals per cage [3]. All females used in experiments were mated. Food was changed every other day. Experimental flies were reared for 15 days in the mortality cages on food with 10% sucrose, 5% yeast, 1.2% agar, and 0.18% methylparaben, and supplemented with 0.04%, 0.12%, 0.4%, or 3% chili (*Capsicum frutescens* L.) powder. The powder was mixed within freshly prepared medium cooled to 70°C. The food of the control group had the same composition as food of the experimental groups but did not contain chili powder.

### 2.2. Resistance to oxidative stress

To determine resistance to oxidative stress, 15 flies were placed in 15 ml glass tubes with a napkin (12 cm × 12 cm) soaked with 1.5 ml of 20 mM menadione sodium bisulfite in 5% sucrose solution [11]. The number of dead flies was recorded every day at 9 AM, 3PM, and 9 PM.

### 2.3. Trolox equivalent antioxidant capacity

One hundred milligrams of dry powdered *Capsicum frutescens* was added to different concentrations of methanol in water (2 mL): 25%, 50%, or 80% (v/v). The mixtures were heated for 60 min at 40°C. The samples were centrifuged for 5 min at 20000×g at 4°C. The supernatants were used for spectrophotometric measurement of antioxidant properties of the powder.

The cation radical 2,2′-azino-bis (3-ethyl benzothiazoline-6-sulfonic acid) (ABTS^•^+) was generated by reacting 7 mmol ABTS^•^+ and 2.45 mmol potassium persulfate via incubation at room temperature (23°C) in the dark for 12–16 h. Subsequently, the ABTS^•^+ solution was diluted to reach an absorbance of 1.000 ± 0.200 at 734 nm. Then, 10 µL of chili powder extracts were mixed with 200 µL of prepared ABTS^•^+ solution. The mixture was shaken at room temperature and the absorbance reading was taken at 734 nm after 6 min.

All measurements were recorded on a microplate reader Synergy H1 (BioTek, Winooski, VT, USA). The standard curve was prepared using different concentrations of Trolox. The results are expressed as Trolox equivalents (TE) per 100 g of the extract (mmol TE/100 g), with values presenting the mean ± standard deviation.

### 2.4. Enzymatic activities and oxidative stress indices

Flies were homogenized using a Potter-Elvehjem glass/glass homogenizer (1:10 w/v) in 50 mM potassium phosphate buffer (pH 7.5) containing 0.5 mM ethylenediaminetetraacetic acid (EDTA) and 1 mM phenylmethylsulfonyl fluoride and centrifuged (16000×g, 15 min, 4°C) in an Eppendorf 5415R centrifuge. Supernatants were collected and used for the determination of enzymatic activities. Total protein content in whole fruit fly bodies was measured by the Bradford method with serum bovine albumin used as the standard. The activities of superoxide dismutase (SOD, EC 1.15.1.1), glucose 6-phosphate dehydrogenase (G6PDH, EC 1.1.1.49), NADP-dependent isocitrate dehydrogenase (IDH, EC 1.1.1.42), and glutathione-*S*-transferase (GST, EC 2.5.1.18) were measured spectrophotometrically as described earlier [12]. Briefly, SOD activity was assayed at wavelength 406 nm by the ability of the enzyme to inhibit oxidation of quercetin by superoxide anion-radical produced in the redox initiator system of *N*,*N*,*N*′,*N*′-tetramethylethylenediamine (TEMED) in an alkaline buffer (30 mM Tris-HCl, 0.5 mM EDTA, 0.8 mM TEMED, 0.05 mM quercetin, pH 10.0). The reaction was conducted for 6–8 different volumes (2–100 μl) of the supernatant obtained by the above method from 40 to 50 flies. One unit of SOD activity was defined as the amount of enzyme (per milligram protein) that inhibits quercetin oxidation by 50% of maximum. The activity of G6PDH was measured in 1 ml of the mixture containing 50 mM KPi buffer (pH 7.5), 0.5 mM EDTA, 5 mM MgCl_2_, 0.2 mM NADP^+^, and 2 mM glucose 6-phosphate at 340 nm by the rate of NADPH formation. The activity of IDH was measured exploring the same principle as for G6PDH and the buffer contained 50 mM KPi buffer (pH 7.5), 2 mM MgCl_2_, 1 mM NADP^+^, and 0.5 mM isocitric acid. The reaction was started by adding 20 μl of the supernatant to 980 μl of the buffer. The extinction coefficient 6.22 mM^−1^ cm^−1^ for NADPH was used. The activity of GST was assayed at 340 nm by the formation of an adduct between reduced glutathione (GSH) and 1-chloro-2,4-dinitrobenzene (CDNB). The reaction mixture contained 50 mM KPi buffer (pH 7.5), 0.5 mM EDTA, 5 mM GSH, 1 mM CDNB, and 1–5 μl of the supernatant in a final volume of 1 ml. The reaction was launched by the sequential addition of CDNB and supernatant. Blanks were measured without CDNB. The extinction coefficient 9.6 mM^−1^ cm^−1^ for 1-*S*-glutathionyl-2,4-dinitrobenzene was used for calculation of the activity.

The activities of catalase, the levels of protein carbonyls and lipid peroxides were assayed as described by Lushchak et al. [13], and aconitase activity was determined as described by Lozinsky et al. [14]. Briefly, catalase activity was measured in a spectrophotometer at 240 nm by a decrease in the concentration of hydrogen peroxide. The extinction coefficient for hydrogen peroxide of 0.0394 mM^−1^ cm^−1^ was used to calculate the activity. Carbonyl derivatives of proteins were detected by the reaction of the derivatives with 2,4-dinitrophenylhydrazine yielding colored hydrazones, whose concentration was quantified spectrophotometrically. The amount of protein carbonyls was evaluated at a wavelength of 370 nm. The molar extinction coefficient of 22 mM^−1^ cm^−1^ for dinitrophenylhydrazones was used for calculations. The values were expressed as nanomoles per milligram of protein. Lipid peroxide (LOOH) content was determined at 580 nm, based on the absorption of light with this wavelength by a complex of ferric iron with xylenol orange (formed in reaction between LOOH, ferrous iron, and xylenol orange). Flies were homogenized with 10 volumes of 96% (vol.) cold (∼5°C) ethanol, centrifuged for 5 min at 13000×g, and supernatants were used for the assay. The levels of lipid hydroperoxides were expressed as cumene hydroperoxide equivalents per gram of wet weight of fruit flies. Aconitase (EC 4.2.1.3) activity was measured at 240 nm by a decrease in concentrations of *cis*-aconitate. The molar extinction coefficient used for calculations was 0.0037 mM^−1^ cm^−1^.

The levels of high- and low-molecular-mass thiol-containing compounds were measured as described by Lushchak et al. [15]. Free thiols were measured spectrophotometrically at 412 nm based on their reaction with 5,5′-dithio-bis (2-nitro) benzoic acid that yields 2-nitro-5-thiobenzoate anion. Total thiol content (the sum of low- and high-molecular-mass thiol-containing compounds) was measured in the supernatants prepared identically to those used for the measurement of enzyme activities. For measurement of low-molecular-mass thiol-containing compounds (LM–SH) content, supernatants were treated with 10% TCA (final concentration), centrifuged for 5 min at 13000×g and the final supernatants were used for the assay. The molar extinction coefficient of 14 mM^−1^ cm^−1^ was used for calculations. The thiol concentrations were expressed as micromoles of SH-groups per gram of fly wet weight.

### 2.5. Statistical analysis

Experimental data are presented as mean ± standard error. Statistical analysis was performed in R, using functions implemented in packages ‘base’, ‘rstatix’, ‘ggplot2’, and ‘ggfortify’. The datasets were compared using a pairwise *t*-test followed by adjustment of *p*-values by the Benjamini-Hochberg procedure [16]. Mortality curves were compared by the log-rank test in the R package ‘survminer’ followed by adjustment of *p*-values by the Benjamini-Hochberg procedure. Differences between sample means and correlations that gave adjusted *p*-values less than 0.05 in the pairwise *t*-test were considered significant.

## 3. Results

**Figure 2.**
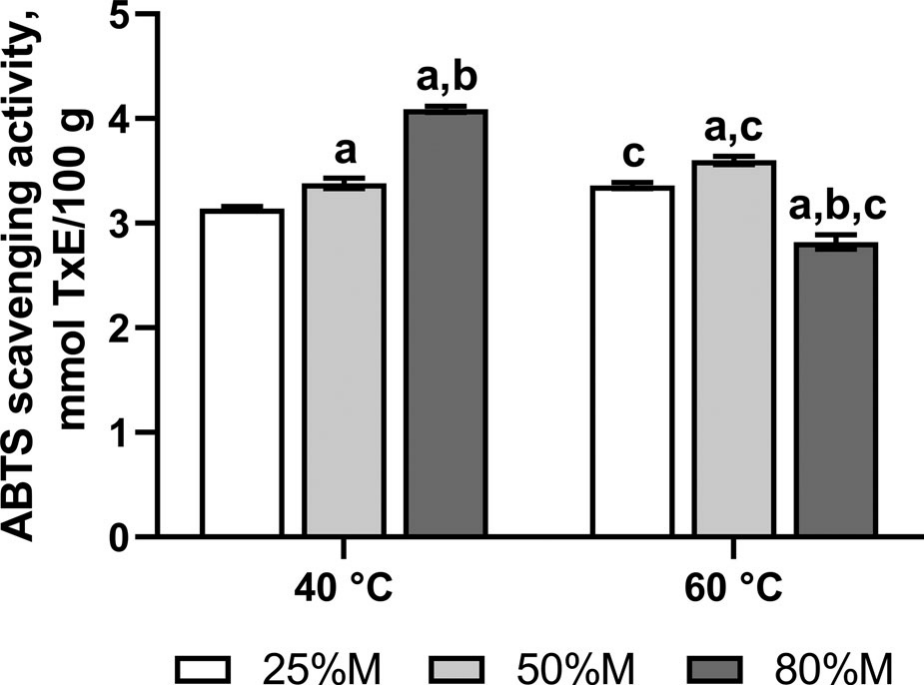
Trolox equivalent antioxidant activity of methanolic extracts of *Capsicum frutescens* powder. M – methanol–water mixtures with indicated concentrations of methanol; TxE – Trolox equivalent. Data are means ± standard deviations (SD), *n* = 4. ^a^The value is significantly different from the values obtained via extraction of polyphenols from dry *Capsicum frutescens* powder with 25% methanol (25%M), ^b^The value is significantly different from the values obtained via extraction with 50% methanol (50%M), ^c^The value is significantly different from the values obtained via extraction by the same concentrations of methanol at 40°C.

Fruit fly cohorts were reared on the medium containing a powder from dry fruits of chili peppers, *Capsicum frutescens*. The chili powder polyphenols exhibited substantial antioxidant activity of about 2.8-4.1 mmol of Trolox (a water-soluble analog of vitamin E) equivalents per 100 g of the powder (Figure 2). The maximum amount of antioxidant compounds was extracted from the powder by 80% methanol at 40°C (Figure 2). However, methanol of the same concentration allowed to extract 31% less antioxidant substances.

Previously, we have shown that consumption of chili-supplemented food extended mean lifespan in *D. melanogaster* cohorts of both sexes by 10%-14%, although effect was more pronounced in females [3]. Significant lifespan extension in males was caused by food supplemented with 0.04% and 0.12% chili powder. In female cohorts, lifespan was prolonged the most by consumption of food supplemented with 0.12% and 0.4% chili powder. On the other hand, consumption of food supplemented with 3% chili powder caused significantly higher mortality among males. Therefore, we chose fruit fly cohorts fed on food with the above concentrations of chili powder for further investigations.

**Figure 3.**
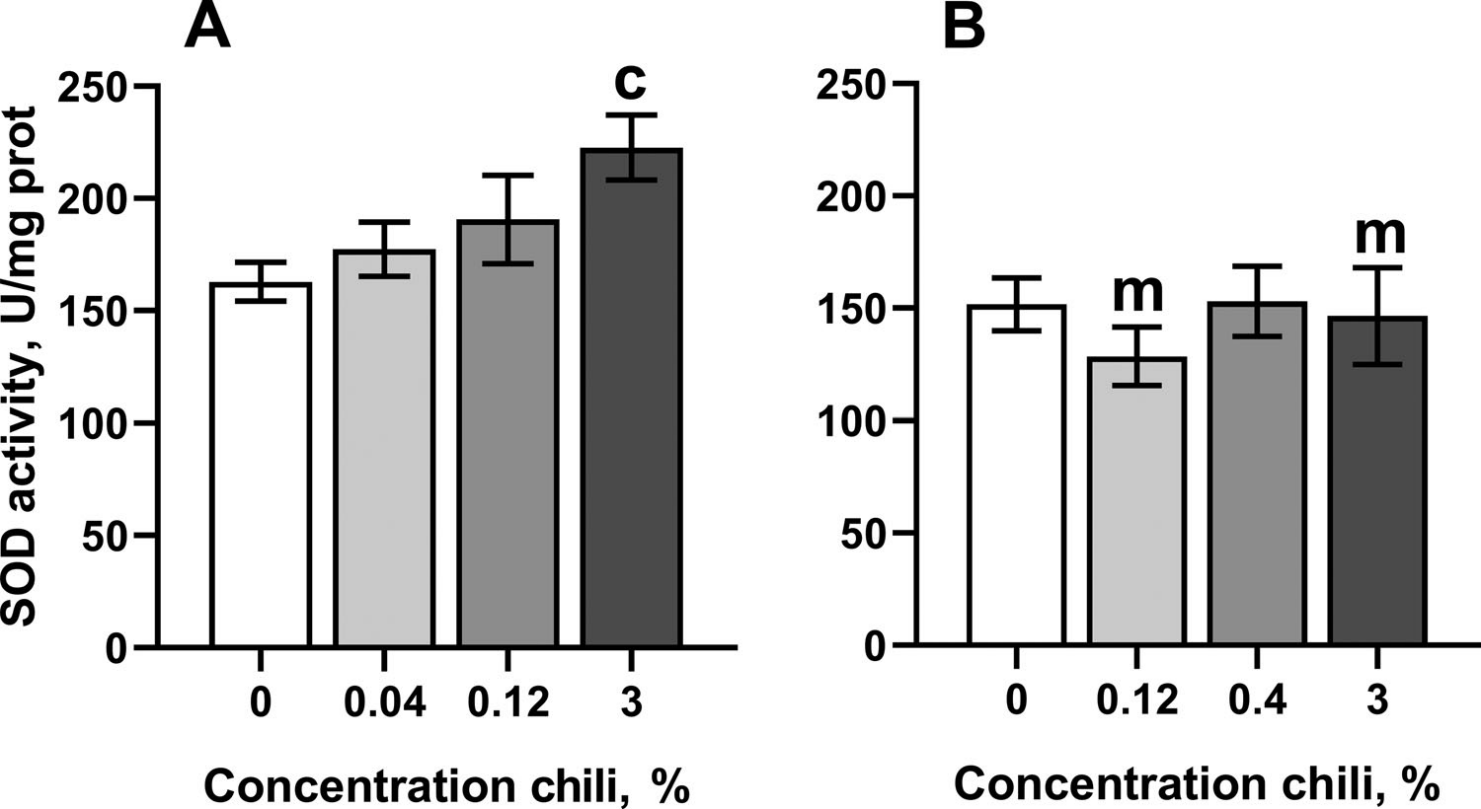
Activity of superoxide dismutase in fruit flies reared for fifteen days on the control diet and the diets supplemented with different concentrations of powder from dry chili fruits: A – males, B – females. Data are means ± SEM (*n* = 4). ^c^Significantly different from the control, *P* < 0.05. ^m^Significantly different from the corresponding group of males, *P* < 0.05.

**Figure 4.**
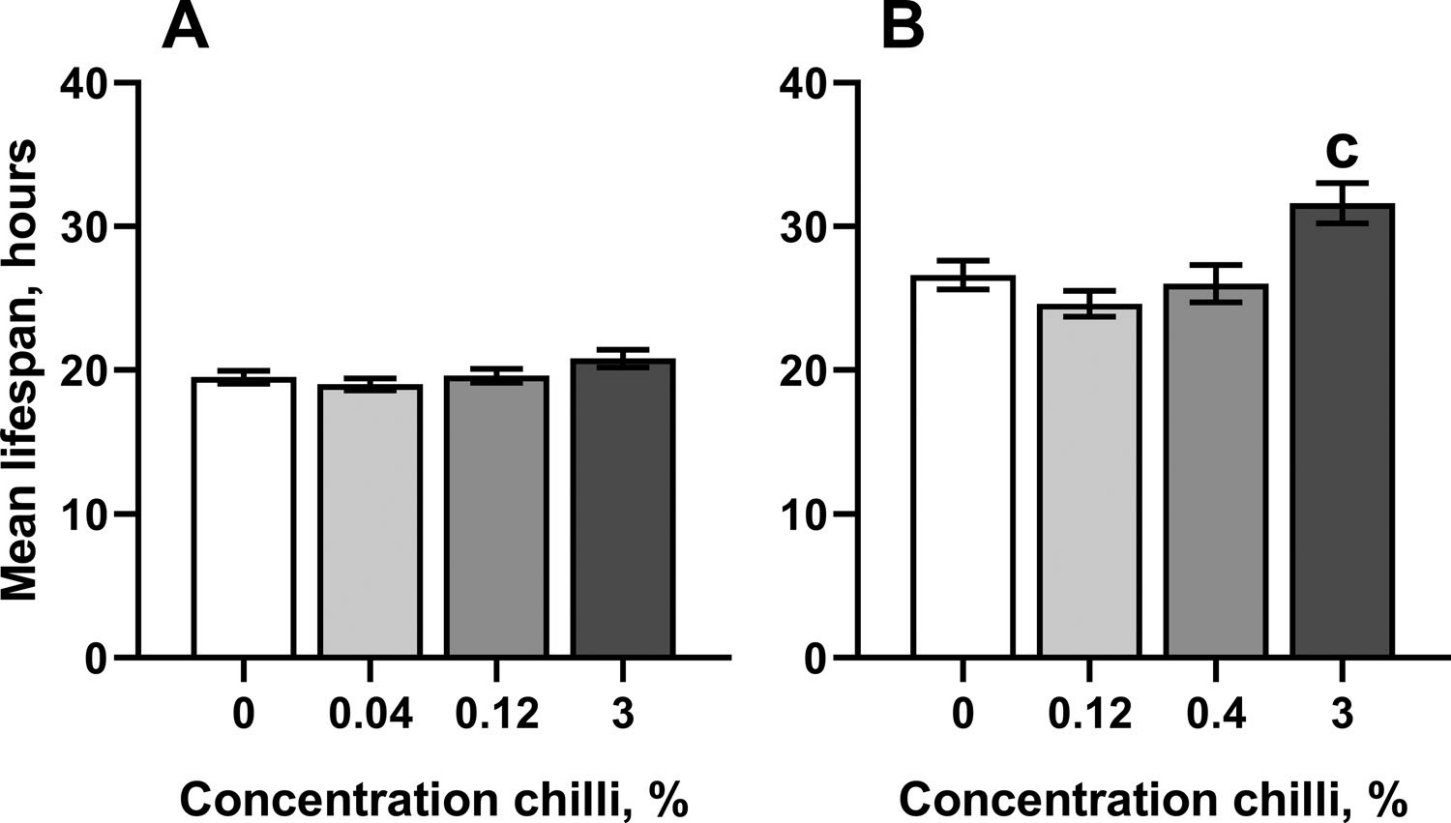
Resistance to redox-cycling compound menadione in fruit flies reared for 15 days on the control diet or the diets supplemented with different concentrations of powder from dry chili fruits: A – males, B – females. Data are means ± SEM (mortality of cohorts of 29–60 individuals was assayed). ^c^Significantly different from the control, *P* < 0.05. Groups were compared using a pairwise log-rank test implemented in R package ‘survminer’ followed by Benjamini-Hochberg correction.

The concentrations of chili powder that prolonged lifespan in *D. melanogaster* weakly affected antioxidant enzymes and markers of oxidative stress. In particular, consumption of food with the highest concentration of chili powder resulted in about 37% higher activity of superoxide dismutase (SOD) in males as compared with the control cohort (Figure 3(A)). However, SOD activity was not substantially affected by consumption of chili-supplemented food in females. Interestingly, females reared on the food with 3% chili powder had slightly higher resistance to the redox-cycling and superoxide-generating compound, menadione, whereas flies of other groups did not differ from the control in their resistance to menadione (Figure 4).

**Figure 5.**
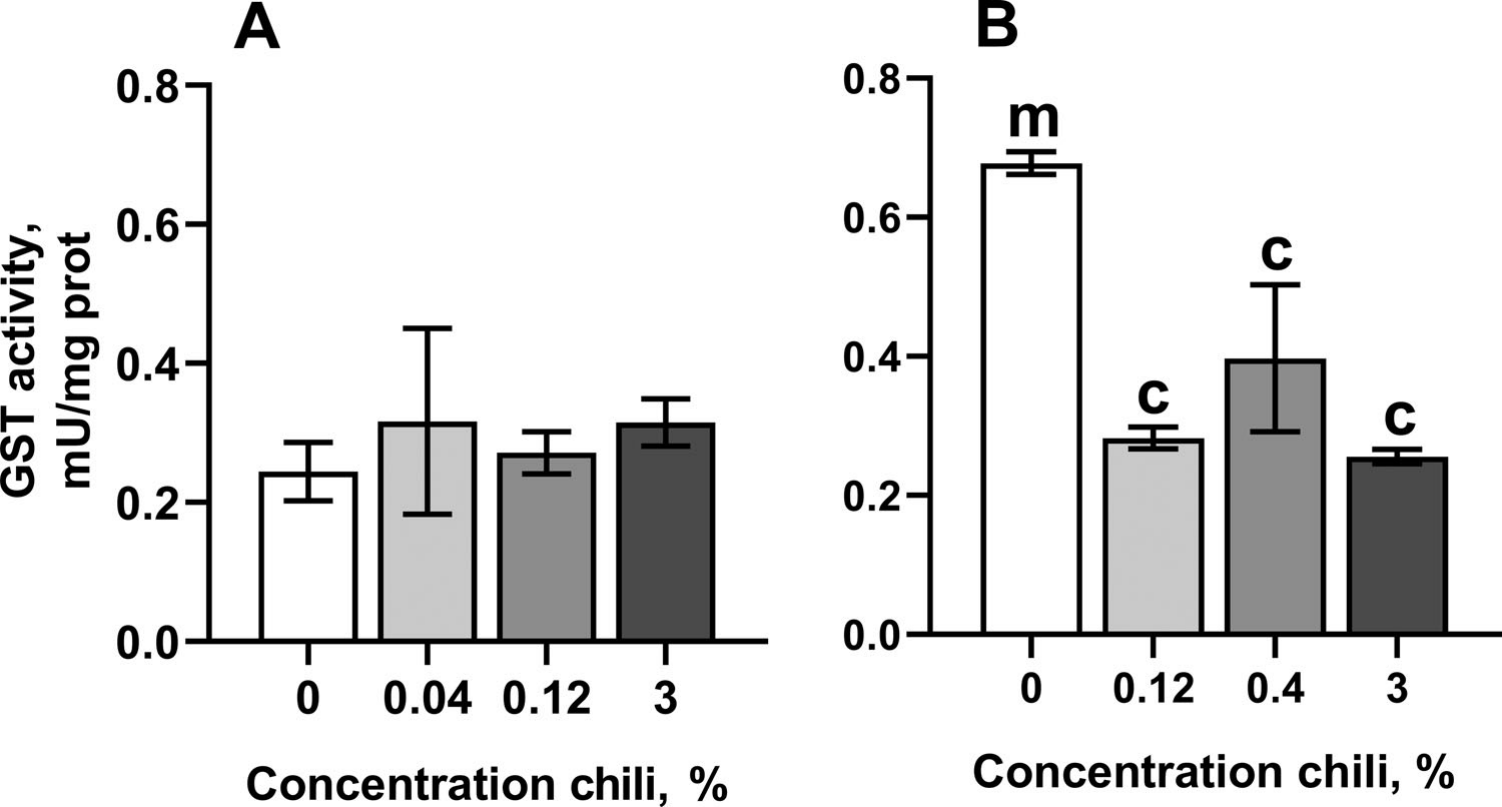
Activity of glutathione-*S*-transferase (GST) in fruit flies reared for fifteen days on the control diet and the diets supplemented with different concentrations of powder from dry chili fruits: A – males, B – females. Data are means ± SEM (*n *= 3–4). ^c^Significantly different from the control, *P* < 0.05. ^m^Significantly different from the corresponding group of males, *P* < 0.05.

The chili-supplemented food did not affect catalase, isocitrate dehydrogenase (IDH), or aconitase activities, and conferred only minor changes on glucose 6-phosphate dehydrogenase activity (G6PDH) (Table 1). Interestingly, consumption of chili-supplemented food led to a significant drop in glutathione-*S*-transferase (GST) activity in females to about 40% to 60% of that in the control cohort (Figure 5(B)). The levels of oxidative stress markers, such as high- and low-molecular mass thiol-containing compounds, protein carbonyls, and lipid peroxides (LOOH), showed only minor changes (Table 1).

**Table 1.** Oxidative stress markers – protein carbonyls (CP), high and low-molecular mass thiols (HM–SH, LM–SH), lipid peroxides (LOOH), and activities of antioxidant and related enzymes: catalase, aconitase, isocitrate dehydrogenase (IDH), and glucose 6-phosphate dehydrogenase (G6PDH) in fruit flies reared for 15 days on the control diet or the diets supplemented with different concentrations of powder from dry *Capsicum frutescens* fruits.

Chili powder, %	0 (Control)	0.04	0.12	3
	Males			
G6PDH, mU/mg prot	44.1 ± 1.7	38.1 ± 1.3	36.6 ± 1.3^c^	41.3 ± 3.6
IDH, mU/mg prot	222 ± 17	215 ± 23	207 ± 19	237 ± 13
Catalase, mU/ mg prot	163 ± 21	189 ± 25	183 ± 24	181 ± 31
Aconitase, mU/mg prot	134 ± 9	134 ± 2	128 ± 4	127 ± 3
LOOH, CHPeqw/gww	4.92 ± 0.43	4.43 ± 0.71	5.74 ± 1.14	5.46 ± 0.61
CP, nmol/mg prot	1.59 ± 0.29	1.45 ± 0.15	1.37 ± 0.37	1.92 ± 0.22
HM-SH, µmol/gww	2.43 ± 0.29	2.19 ± 0.24	2.83 ± 0.55	2.76 ± 0.19
LM-SH, µmol/gww	1.06 ± 0.03	1.26 ± 0.18	1.08 ± 0.01	1.09 ± 0.07
	Females			
Chili powder, %	0 (Control)	0.12	0.4	3
G6PDH, mU/mg prot	21.0 ± 1.3^m^	24.8 ± 1.4^m^	26.1 ± 2.4	23.1 ± 2.4^m^
IDH, mU/mg prot	160 ± 8^m^	175 ± 7	159 ± 13	156 ± 16^m^
Catalase, mU/ mg prot	73 ± 4^m^	79 ± 6^m^	86 ± 7	72 ± 10^m^
Aconitase, mU/mg prot	103 ± 2^m^	106 ± 1	115 ± 7	115 ± 14
LOOH, CHPeqw/gww	4.68 ± 0.38	5.00 ± 0.54	3.48 ± 0.21	4.05 ± 0.25^m^
CP, nmol/mg prot	2.27 ± 0.24	2.78 ± 0.52^m^	2.45 ± 0.32	2.00 ± 0.47
HM-SH, µmol/gww	2.88 ± 0.27	2.46 ± 0.12	3.30 ± 0.19	3.10 ± 0.90
LM-SH, µmol/gww	1.07 ± 0.03	1.17 ± 0.08	1.07 ± 0.04	1.16 ± 0.03

Data are means ± standard errors of the means (SEM), *n* = 3–8.

^c^ Significantly different from the control, *P* < 0.05.

^m^ Significantly different from the corresponding group of males, *P* < 0.05.

**Figure 6.**
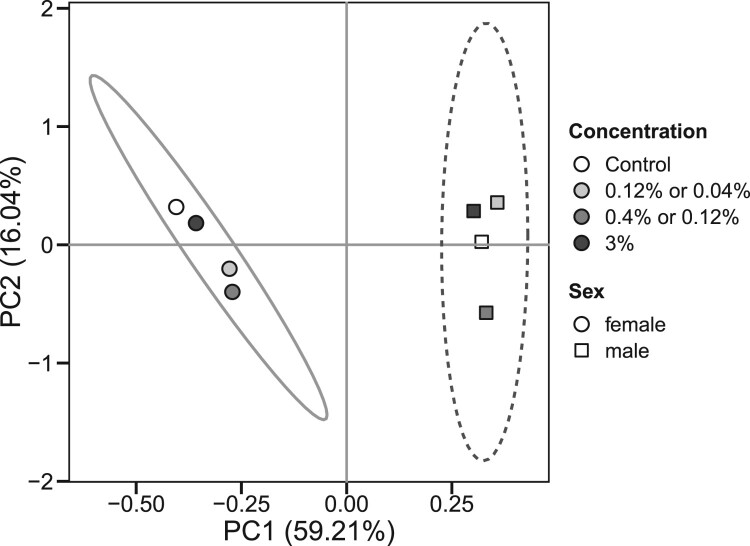
Principal component analysis of the biochemical parameters (SOD, catalase, GST, G6PDH, IDH, aconitase, high- and low-molecular mass thiols, CP, LOOH) measured in fruit flies reared for 15 days on the control diet or diets supplemented with different concentrations of powder from dry chili fruits. Each point (circle or square) represents an average for a male/female fruit fly cohort reared on either control food or on food with indicated concentration of chili powder. Circles denote averages for female cohorts whereas squares denote averages for male cohorts. Coordinates of each point on the plot are determined by the average values of the above-mentioned biochemical parameters that were converted into the loadings of principal components. Ellipses indicate 68% confidence intervals for the data. Percentages at principle component axes indicate the amounts of variance explained by each principal component.

**Figure 7.**
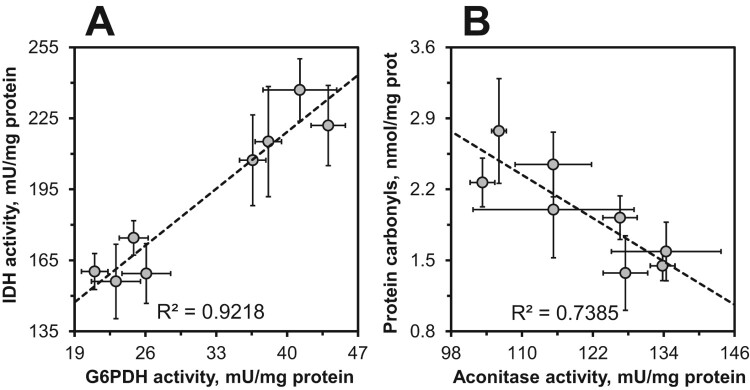
Linear correlations between G6PDH and IDH (A), and between aconitase and protein carbonyl content (B) in *D. melanogaster* reared on the control diet and the diets supplemented with different concentrations of chili powder.

It is remarkable that nearly all parameters studied showed dependence on fly sex that in many cases was not significantly affected by consumption of chili-supplemented food. This sex dependence was observed for catalase, G6PDH, IDH, GST, aconitase, and protein carbonyls. A strong sex dependence of antioxidant defenses in general was also found by the principal component analysis (Figure 6). The sex dependence along with coordinated minor changes in some parameters resulted in strong significant correlations between some parameters. In particular, strong correlations were found between SOD and catalase, catalase and G6PDH, IDH and catalase, as well as between IDH and SOD (Table 2). Strong correlations were also observed between G6PDH and IDH (Figure 7(A)), aconitase and catalase, G6PDH, and IDH, as well as between protein carbonyls and aconitase (Figure 7(B)), catalase, and G6PDH.

**Table 2.** Pearson correlation coefficients between all measured biochemical parameters.

	SOD	CAT	GST	G6PDH	IDH	ACO	HM–SH	LM–SH	CP	LOOH
SOD	–	0.813*	–0.166	0.718	0.796*	0.636	–0.102	–0.150	–0.617	0.542
CAT	–	–	–0.424	0.928*	0.936*	0.900*	–0.534	0.100	0.824*	0.577
GST	–	–	–	–0.522	–0.436	–0.592	0.273	–0.283	0.341	–0.199
G6PDH	–	–	–	–	0.960*	0.925*	–0.535	–0.046	0.729*	0.512
IDH	–	–	–	–	–	0.827*	–0.604	0.024	–0.663	0.654
ACO	–	–	–	–	–	–	–0.452	0.128	0.858*	0.277
HM–SH	–	–	–	–	–	–	–	–0.535	0.344	–0.430
LM–SH	–	–	–	–	–	–	–	–	–0.088	–0.178
CP	–	–	–	–	–	–	–	–	–	–0.378

Asterisk denotes statistically significant correlations.

## 4. Discussion

Capsaicin, a constituent of chili pepper, was found to prolong lifespan in the fruit fly *Drosophila melanogaster* [2]. Our recent study has revealed that fruit flies reared on the food supplemented with chili powder live longer than the counterparts on the control diet [3]. The effect can be accounted for by capsaicin as well as by other phenolic substances in chili pepper.

As we demonstrated in the recent study, the maximum amount of phenolic compounds that could be extracted by 80% methanol at 40°C from the powder we use was 942 mg of gallic acid equivalents per gram of the preparation [3]. Current data (Figure 2) show that Trolox equivalent antioxidant capacity of different methanolic extracts of our chili powder correlates with the amount of polyphenols present in the powder. It is comparable with the data obtained in other studies and indicates a moderate amount of antioxidant substances [17]. Chili powder is rich in carotenoids that are established antioxidants. However, trolox antioxidant capacity is likely related to the amount of polyphenols present in the powder since often these parameters positively correlate [18].

The maximum amounts of capsaicinoids – capsaicin, dehydrocapsaicin, and nordihydrocapsaicin – could be extracted at the same extraction conditions [3]. Mixing dry powder with a hot (approximately 70°C) fruit fly medium allows only partial extraction of phenolic compounds and capsaicinoids. It was shown that extraction with hot water gave 4–6 times smaller amounts of extracted capsaicinoids than it could be achieved with methanol [19,20].

Of note, our previous study showed flies reared on the control medium and on chili-supplemented food consumed approximately equal amounts of meal – 40–60 nl/fly/hour [3]. However, female cohort on the medium with 3% chili powder consumed on average twice as higher amount of medium than counterparts on the control medium [3].

The extension of lifespan in fruit fly cohorts by regular consumption of chili-supplemented food can be conferred by the activation of well-known pro-survival pathways. In particular, a number of plant preparations activate the transcription factor FOXO (forkhead box O), increasing the expression of proteins that are necessary to overcome stress conditions [1,21]. Lifespan extension is also achieved by the inhibition of mTOR (mechanistic target of rapamycin) kinase, that activates autophagy [22]. At the same time, it is known that many life-prolonging plant preparations directly or indirectly affect antioxidant responses [1,6]. Antioxidant defenses could be activated by the phenolic compounds of chili pepper via the transcription factor Nrf2 (nuclear factor-erythroid factor 2-related factor 2). However, our current study shows that chili-supplemented food affects only two antioxidant enzymes, namely superoxide dismutase (SOD) in males (Figure 3) and glutathione-*S*-transferase (GST) in females (Figure 5). Whereas SOD activity was boosted in male cohorts reared on food with 3% chili powder, GST activity was decreased in females on all chili-supplemented diets. It is worth noting, that 3% chili was previously found to be a rather life-shortening concentration of chili powder [3]. Overexpression of SOD was found to prolong lifespan in fruit flies [8,23–25]. However, there is a controversy regarding life-prolonging effects of SOD in other models [8]. A higher SOD activity makes sense only with a concomitant increase in catalase activity. The reason for this is that the product of the SOD reaction is hydrogen peroxide, a type of ROS, which participates in the Fenton reaction, yielding hydroxyl radical [26,27]. In turn, hydroxyl radicals are strong oxidizers, that react with proteins and fatty acids, causing loss of function to proteins and lipid membranes, respectively [26]. We cannot draw a direct connection between consumption of chili-supplemented food, SOD activity, and lifespan in *D. melanogaster*. Likely, capsaicin and/or bioactive phenol-containing compounds of chili powder affect signaling pathways that regulate SOD activity on transcriptional or posttranslational levels. The appearance of the effect only in males may imply that the signaling pathway plays an important role for this gender. Genes that encode antioxidant enzymes, including SOD, are targets for the transcription factors FOXO and Nrf2 in mammals [21,28,29]. However, much less is known about regulation of antioxidant enzyme expression in fruit flies. Earlier, it was found that SOD expression is indirectly suppressed by a dual-specificity kinase Doa (darkener of apricot) in *D. melanogaster* as well as in human cultured cells [30]. The activity of SOD increased in flies with mutated Doa. In turn, it was found that the LAMMER kinase, homologous to Doa, is activated by mTOR kinase in the budding yeast, *Saccharomyces cerevisiae* [31]. In addition, it was found that Doa plays role in sex determination, affecting the production of sex pheromones and courtship behavior [32].

We expected to see higher activities of all antioxidant enzymes in flies reared on chili-supplemented food. However, activities of many enzymes that we studied showed only a subtle difference in flies that consumed chili-supplemented food as compared with controls. Moreover, GST activity was lower in females fed on food containing chili powder than in control females. Most of genes that encode different isoenzymes of GST in *D. melanogaster* are regulated by Nrf2 as well as by nuclear receptors such as DHR96 [33–35] or Seven-up [36]. In turn, flavonols, such as kaempferol, are able to inhibit nuclear receptors [37]. Moreover, nuclear receptors are expressed in sex-specific manner, respond to sex-specific steroid hormones, and exert sex-specific effects [36,38,39]. It was demonstrated that a number of GST isoenzymes are regulated in a sex-dependent manner in *D. melanogaster* [40]. This may explain the female-specific response of GST activity to a regular consumption of chili-supplemented food.

**Figure 8.**
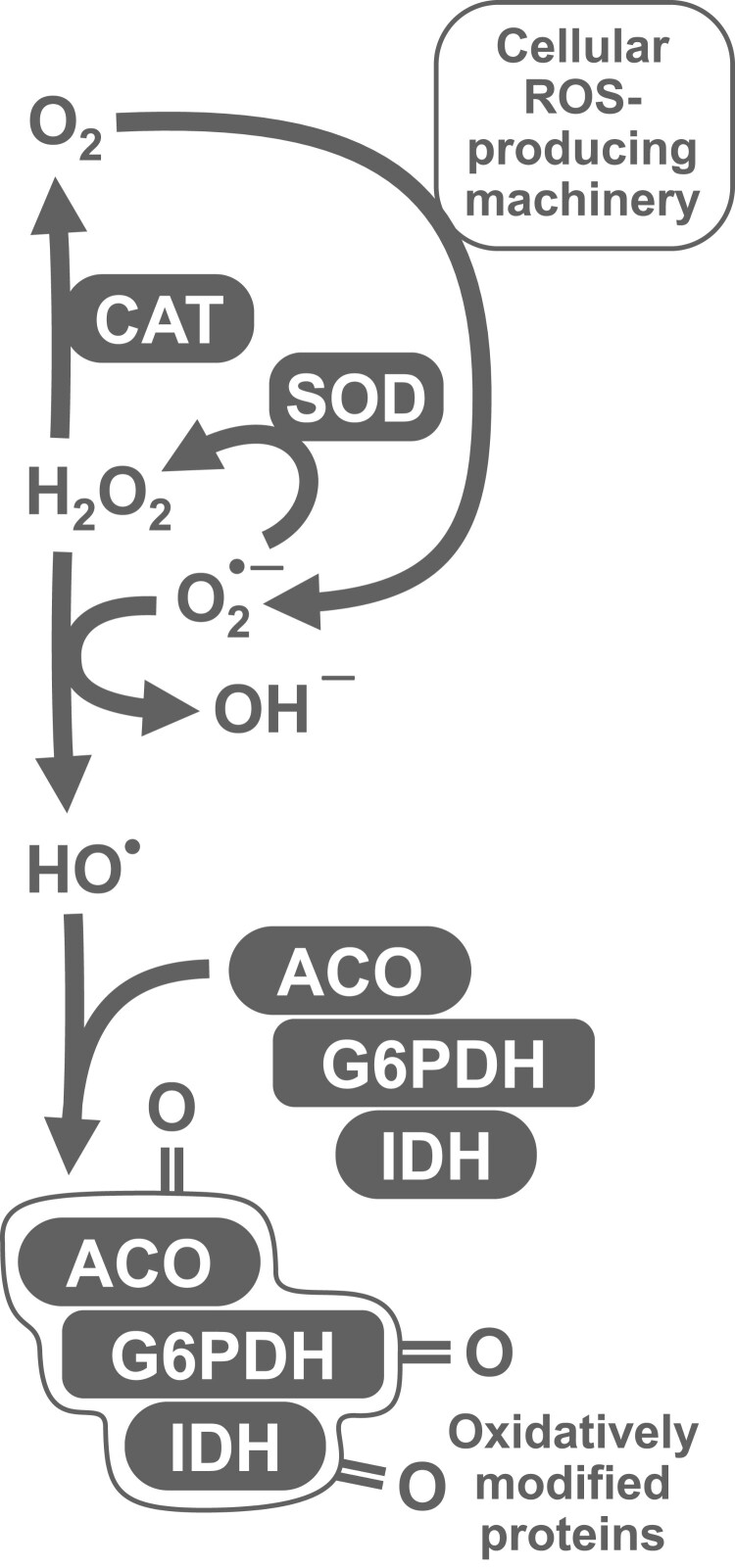
Generalized scheme that explains relationships between antioxidant (SOD and catalase) and related enzymes (G6PDH, IDH), and potential oxidative stress markers (protein carbonyls) observed in the study and partially confirmed by regression analysis. SOD converts superoxide radical into less toxic hydrogen peroxide. However, SOD protects biomolecules from oxidation by ROS only in conjunction with catalase, since the latter prevents potential formation of hydroxyl radicals in the reaction between superoxide and hydrogen peroxide. G6PDH, IDH, and aconitase were shown to be sensitive to oxidative modification, and therefore can be oxidized by hydroxyl radicals and contribute to the pool of carbonylated proteins.

Our study also shows a pronounced sex dependence of antioxidant defenses in *D. melanogaster*. We have noticed this feature in our previous studies, finding differences between males and females in activities of catalase and G6PDH, as well as in the levels of protein thiols [12,13,15]. Our current data also show a sex bias in the activities of IDH, GST, SOD, and aconitase. A higher catalase activity in males was also reported by other researchers [41,42], although the opposite situation was also observed [43]. The gene that encodes G6PDH, is located on the X-chromosome. The higher G6PDH activity in males can be explained by over-compensation of gene dosage [44,45]. Other cytosolic enzymes, such as NADP-dependent isocitrate dehydrogenase and cytosolic aconitase (also known as iron regulatory protein), are not directly associated with the X-chromosome. However, integrity of these enzymes may depend on the sex-linked enzymes such as catalase and G6PDH. The set of correlations (Table 2) and principal component analysis (Figure 6) provide grounds for such dependence. In particular, we see strong correlations between all sex-linked indices of our study (Table 2). In turn, oxidative stress indices such as LOOH, protein thiols, and low-molecular mass thiol-containing compounds did not show correlations with other parameters (Table 2). Hence, most significant correlations were conferred by a linkage of measured parameters with fly gender. Nevertheless, the observed correlations seem logical, as we explained in our previous studies [15,46–49]. In particular, G6PDH and IDH activities correlated with catalase activity and that may imply a role of catalase in the protection of these enzymes from oxidative modification [15,46–49]. Alternatively, these enzymes could be co-regulated with catalase and be targets of the same transcriptional regulator. Aconitase, as an enzyme that contains iron-sulfur clusters (sensitive to oxidation), is also a well-established marker of oxidative stress [10]. Along with IDH and G6PDH, it can also be oxidatively modified by ROS or protected by the first-line antioxidant enzymes, SOD and catalase (Figure 8). On the other hand, operation of G6PDH and IDH maintains NADPH pools and, in turn, NADPH is required for the assembly of iron-sulfur clusters [50,51].

We conclude that regular consumption of chili-supplemented food extends lifespan in fruit fly cohorts in a concentration- and gender-dependent manner. However, this extension is not mediated by a strengthening of antioxidant defenses. Fruit flies that consumed chili-supplemented food show differences only in activities of SOD and GST. The minor changes observed imply that the effect of chili on lifespan has a weak connection with antioxidant defense. Furthermore, consumption of chili-supplemented food does not change the specific relationship between antioxidant and related enzymes in *D. melanogaster*, and also does not change the linkage of the activities of these enzymes to fly gender.

## Data Availability

The findings of this study are available from the corresponding author upon request.
